# SARC-F Capacity to Tracking Sarcopenia in Women From Manaus-Am

**DOI:** 10.1093/geroni/igaa057.472

**Published:** 2020-12-16

**Authors:** Myrian Faber, Fatima Baptista

**Affiliations:** 1 University of the State of Amazonas, MANAUS, Amazonas, Brazil; 2 Faculty of Human Motricity, University of Lisbon, Faculty of Human Motricity, University of Lisbon, Lisboa, Portugal

## Abstract

This study aimed to analyze the performance of SARC-F for the identification of sarcopenia in women from Manaus. For the analysis of the performance of the SARC-F, the criteria defined by the EWGSOP (2018) and the SDOC (2019) were considered. The sample consisted of 236 women aged 66.27±5.76 years. In addition to the SARC-F, a dynamometer was used to assess the muscle strength and gait speed was determined on a 2.44 m course. To identify sarcopenia, the following cut-off valueswere used: SARC-F ≥ 4 points; grip strength: EWGSOP <27kg for men and 16kg for women, SDOC <35.5kg for men and <20.0 kg for women; grip over body mass index (BMI, kg/m2) <1.05 for men and 0.79 for women; grip over body weight (BW, kg) <0.45 for men and <0.34 for women; gait speed ≤ 0.8 m/s for both men and women. The results revealed a prevalence of sarcopenia in 54% of the sample. The Kappa statistic intended to analyze the agreement between the SARC-F and the grip strength, the grip strength corrected for BMI or for BM, and the gait speed. The cross-classification analysis showed linear weighted Kappa coefficients near 0 with exception of gait speed (0.264±0.054, agreement in 61.2% of the participants) and grip strength when the cut off is 20 kg (0.248±0.062 - agreement in 63% of the participants). Cross-classification analysis between SARC-F and objective measures of physical capacity (grip strength and gait speed) showed linear weighted Kappa coefficients with slight or fair agreement in women from Manaus.

